# Profiling of the Endogenous Phenolic Contents of Multifloral Honey From Different Geographical Origins in Türkiye by LC‐MS/MS

**DOI:** 10.1002/fsn3.71555

**Published:** 2026-02-19

**Authors:** Nilgün Paksoy, Hisamettin Durmaz, Serap Kılıç Altun, Mehmet Emin Aydemir

**Affiliations:** ^1^ Department of Biochemistry, Faculty of Veterinary Medicine The University of Harran Şanlıurfa Türkiye; ^2^ Department of Food Hygiene and Technology, Faculty of Veterinary Medicine The University of Harran Şanlıurfa Türkiye

**Keywords:** bee, honey, LC‐MS/MS, phenolic content, Türkiye

## Abstract

Honey is an important animal product. It has been known about since ancient times. It is still a matter of curiosity today. This is due to its extensive biological properties and characteristic chemical composition. This study aimed to determine the phenolic content of honey from various regions of Türkiye. A total of 99 multifloral honey samples were collected from beekeepers in various Turkish provinces, and 25 phenolic compounds were analyzed using liquid chromatography‐tandem mass spectrometry (LC‐MS/MS). The highest measured phenolic component was hydroxycinnamic acid in honey samples from the Eastern Anatolia (103.60 ± 17.76 μg/100 g) and Central Anatolia (100.94 ± 12.79 μg/100 g) regions, followed by the Marmara (119.32 ± 17.26 μg/100 g), Aegean (147.83 ± 15.99 μg/100 g), and the Black Sea region (204.83 ± 33.28 μg/100 g). The most abundant phenolic component of the Mediterranean Region (114.38 ± 41.52 μg/100 g) and Southeastern Anatolia Region (148.53 ± 81.72 μg/100 g) honeys was vanillic acid. Alizarin, myricetin, protocatechuic acid, thymoquinone, and 2‐hydroxy‐1,4‐naphthoquinone were not detected among these phenolic compounds in the honey samples. These results emphasize the effect of geographical differences and the flora from which honey is derived on its phenolic content once again. They also provide valuable information about the phenolic content of honey produced in different regions of Türkiye, which has a wide variety of flora and honey types.

## Graphical abstract

This study investigated the phenolic profiles of 99 multifloral honey samples collected from seven geographical regions of Türkiye using LC‐MS/MS. Significant regional differences were observed in the concentration and diversity of 25 phenolic compounds, with hydroxycinnamic acid and vanillic acid being the most prevalent. The results highlight the influence of geographical origin on honey composition and support phenolic profiling as a tool for honey authentication.

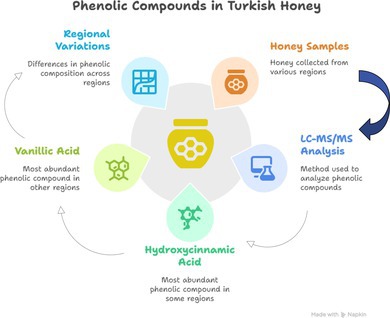

## 1 Introduction

Bioactive compounds of natural origin have attracted a great deal of interest in recent years. While the health benefits of natural products are widely recognized as superior to those of synthetic products, ongoing discussions and studies are exploring this issue (Topliss et al. 2002). Natural products such as honey and herbs have long been an integral part of folk medicine practices around the world, highlighting the importance of understanding their biological origins in relation to human well‐being (Kaškonienė et al. 2009).

Honey is one of the oldest and most traditional sweetening agents, consisting of approximately 200 different substances (Gheldof et al. 2002). These include enzymes, flavonoids, phenolic acids, volatile compounds, and various sugars, such as glucose, fructose, and sucrose. Honey also contains proteins (approximately 0.5%), moisture (approximately 17.5%), and vitamins and minerals (typically 0.04%–0.2%). The main components of honey are water, glucose, fructose, sucrose, minerals, and proteins (Al‐Mamary et al. 2002). Honey, a substance produced by bees, has been highly valued throughout human history for its wide‐ranging medicinal and nutritional benefits (Kolaylı et al. 2023). Due to its varied composition, influenced by factors such as the types of flowers visited by the bees, its geographical origin, and processing methods, honey is a subject of continuous research by scientists (Machado De‐Melo et al. 2018; Uçurum et al. 2024). A particular focus of scientific research has been its phenolic compounds, which are renowned for their potent antioxidant, antimicrobial, and anti‐inflammatory properties. It is believed that these bioactive components contribute significantly to the health‐enhancing effects observed with regular honey consumption (Cianciosi et al. 2018).

Phenolic compounds are a key group of secondary metabolites found in honey, influencing its sensory appeal and health properties (Da Silva et al. 2016). These compounds, which include flavonoids, phenolic acids, and polyphenols, originate from a variety of floral sources and significantly contribute to honey's antioxidant capabilities, combatting oxidative stress (Crozier et al. 2009). They also exhibit antimicrobial properties, which aid honey preservation and prevent spoilage (Estevinho et al. 2008). Furthermore, phenolic compounds possess anti‐inflammatory properties, enhancing honey's potential to treat inflammation‐related conditions. Their presence is associated with honey's reputed health benefits in combatting cancer, diabetes, and cardiovascular diseases (Cianciosi et al. 2018; Ranneh et al. 2021).

However, the composition of phenolic compounds in honey can vary significantly depending on factors such as the floral source, seasonal and environmental conditions, geographical origin, and processing methods (Joshi et al. 2000). This variability results in distinct chemical compositions, physical properties, taste profiles, and biological activities among different honeys worldwide. Moreover, the phenolic composition can be used to assess honey quality and authenticity, distinguishing between monofloral and multifloral varieties and providing information about their origins (Becerril‐Sánchez et al. 2021). This highlights the importance of phenolic compounds when evaluating honey as a functional food and natural remedy (Luchese et al. 2017).

With its diverse vegetation and unique geography, Türkiye offers an interesting area for the study of honey phenolics, which are substances found in honey that are produced by the action of bees on nectar. Multifloral honey, produced from a variety of plant sources, contains a complex matrix with high phenolic diversity (Can et al. 2015). Studies conducted on the phenolic substance content of Turkish honey over the last decade have mostly focused on provincial, regional and uniflora honey samples (Can et al. 2015; Çayan et al. 2023; Uçurum et al. 2024; Kolaylı et al. 2024). This study aimed to investigate 25 different phenolic compounds in 99 multifloral honey samples taken from 34 provinces in seven regions across Türkiye, using advanced analytical techniques such as liquid chromatography and tandem mass spectrometry (LC‐MS/MS). The aim was to characterize the phenolic profile of Turkish multifloral honey, reveal its nutritional and medicinal benefits, and improve our understanding of its potential health effects. This research could inform quality control measures in the honey industry, ensuring the authenticity and standardization of Turkish multifloral honey products. In addition to improving our understanding of the phenolic components of Turkish honey, this research can also support the value of honey in global markets.

## 2 Materials and Methods

### 2.1 Sample Collection

**FIGURE 1:**
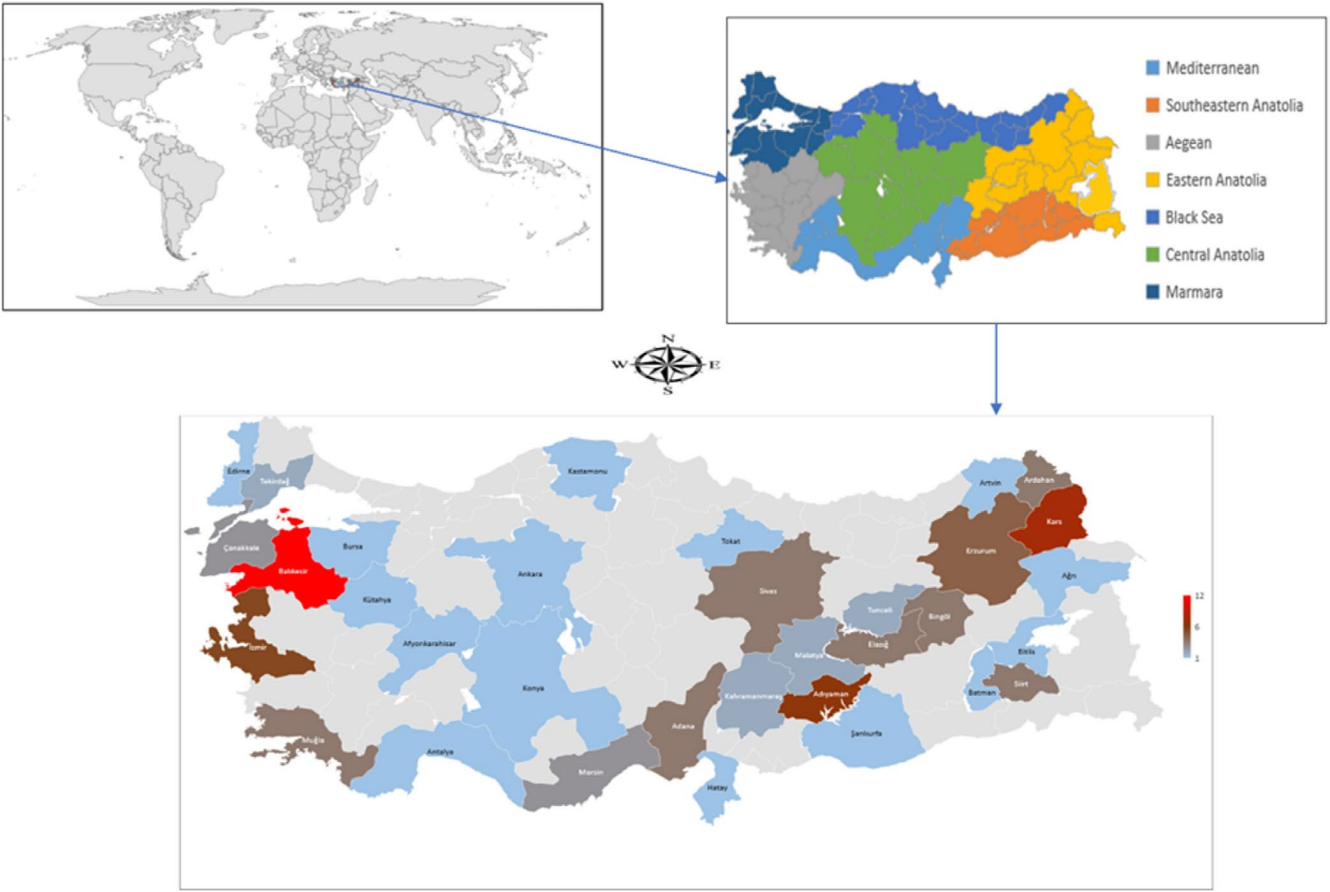
A map of Türkiye showing the regions where honey samples were collected.

A total of 99 multifloral honey samples harvested between March and August 2018 were purchased directly from beekeepers from seven different geographical regions of Türkiye. 50 g of honey from each honey sample was transported to the laboratory in sterile polyethylene tubes and stored in the dark at room temperature until analyzed. The geographical origin of the total 99 honey samples that constitute the material of the study was presented in Figure 1 and Table 1.

**TABLE 1:** The geographical origin of honey samples.

Regions	Eastern Anatolia (*n*: 35)	Mediterranean (*n*: 11)	Marmara (*n*: 19)	Black Sea (*n*: 3)	Aegean (*n*: 11)	Central Anatolia (*n*: 7)	Southeastern Anatolia (*n*: 13)
Provinces	Ağrı (*n*: 1)	Adana (*n*: 4)	Balıkesir (*n*: 12)	Artvin (*n*: 1)	İzmir (*n*: 6)	Ankara (*n*: 1)	Adıyaman (*n*: 7)
	Ardahan (*n*: 4)	Antalya (*n*: 1)	Bursa (*n*: 1)	Kastamonu (*n*: 1)	Kütahya (*n*: 1)	Afyon (*n*: 1)	Batman (*n*: 1)
	Bitlis (*n*: 1)	Hatay (*n*: 1)	Çanakkale (*n*: 3)	Tokat (*n*: 1)	Muğla (*n*: 4)	Konya (*n*: 1)	Siirt (*n*: 4)
	Bingöl (*n*: 4)	Kahramanmaraş (*n*: 2)	Edirne (*n*: 1)			Sivas (*n*: 4)	Şanlıurfa (*n*: 1)
	Elazığ (*n*: 4)	Mersin (*n*: 3)	Tekirdağ (*n*: 2)				
	Erzurum (*n*: 5)						
	Kars (*n*: 8)						
	Malatya (*n*: 2)						
	Tunceli (*n*: 2)						
	Van (*n*: 4)						

### 2.2 Honey Extraction Procedure

The LC‐MS/MS analysis extraction method was adapted from that outlined by Durmaz (2020) and originally described by Altun and Aydemir (2020). All chemicals used were of analytical grade and were purchased from Merck in Darmstadt, Germany. To prepare the samples, 25 g of honey was thoroughly mixed with acidic water (pH 2, using HCl) at a ratio of 5:1 and filtered through cotton to remove any solid particles. The resulting filtrate was passed through a glass column packed with Amberlit XAD‐4 resin, which selectively retained the phenolic compounds while allowing the sugars and other polar substances to be washed away with an aqueous solution. After washing the column with acidic water and purified water, the phenolic fraction was concentrated using methanol and reduced pressure. The remaining residue was dissolved in water, partitioned with diethyl ether and then combined and concentrated using a rotary evaporator. Since many studies have focused on extracting phenolic compounds from honey using Amberlite XAD‐4 resin, and since this method requires the use of diethyl ether in addition to a significant amount of honey and solvent, diethyl ether was preferred in this study because of its selective extraction capability (Gheldof et al. 2002; Kenjerić et al. 2007; Iurlina et al. 2009; Bertoncelj et al. 2011).

### 2.3 Analysis of Phenolic Composition by LC–MS/MS

For the LC‐MS/MS analysis, the concentrated residue was dissolved in methanol and filtered through a 0.45 μm membrane before being analyzed for phenolic substances using a UHPLC instrument coupled with a tandem MS device. This study investigated the presence of the following phenolic compounds (catechin hydrate, acetohydroxamic acid, vanillic acid, resveratrol, fumaric acid, gallic acid, caffeic acid, phlorizin dihydrate, oleuropein, hydroxycinnamic acid, ellagic acid, myricetin, protocatechuic acid, silymarin, 2‐hydroxy‐1,4‐naphthoquinone, butein, naringenin, luteolin, kaempferol, curcumin, thymoquinone, alizarin, hydroxybenzoic acid, salicylic acid and quercetin) in multifloral honeys. The LC–MS/MS system consists of a UHPLC Shimadzu Nexera device coupled with a triple quadrupole mass spectrometer. This system comprises an LC‐20 AD gradient pump, a DGU‐20A3R degasser, a CTO‐10ASvp column oven, and a SIL‐20 AC autosampler. Chromatographic separation was conducted using a C‐18 Intersil ODS‐4 column (3.0 mm × 100 mm, 2 μm). The column temperature was 40°C and the injection volume at 2 μL. The mobile phase was composed of 0.1% formic acid in ultrapure water (solvent A) and 0.1% formic acid in methanol (solvent B). The flow rate of the mobile phase was set at 0.3 mL/min. Chromatographic separation was conducted with a total run time of 10 min. The mobile phase was initially composed of 95% solvent A and 5% solvent B, and a linear gradient was applied between 0 and 4 min to reach 95% solvent B. The system was then held isocratically at 95% solvent B from 4 to 7 min to elute less polar compounds and facilitate column washing. At 7.01 min, the mobile phase returned to initial condition (95% solvent A and 5% solvent B), and the system was maintained at these conditions for the remainder of the run to ensure complete re‐equilibration of the column for the subsequent injection.

Mass spectrometric analyses were performed using a triple quadrupole mass spectrometer with an electrospray ionization (ESI) interface. The MS was operated in scheduled multiple reaction monitoring (MRM) mode. To ensure stable ion production in both negative and positive ionization modes, the ESI source and gas parameters were maintained as follows: nebulizing gas flow: 3.0 L/min (nitrogen), drying gas flow: 15.0 L/min (nitrogen), heating gas flow: 10.0 L/min (air), collision gas (argon): 230 kPa, desolvation line temperature: 250°C, heat block temperature: 400°C.

Optimized MRM transitions, retention times, and validation parameters for the pfenolic compounds analyzed by LC‐MS/MS are presented in Table S1.

### 2.4 Statistical Analyses

Statistical analyses were performed using IBM SPSS Version 16.00 (SPSS Inc., Chicago, IL, USA) software. One‐way analysis of variance (ANOVA) was used to compare the levels of phenolic compounds in honey from each region. Following the variance analysis, a Duncan's multiple range test was performed to determine the differences between the group means. The results are presented as the mean ± standard error (IBM SPSS 2017). PCA statistical analysis was performed using Minitab 16.2.1 software (Minitab Inc 2010). Prior to PCA, all variables were standardized to have a mean of zero and a variance of one.

## 3 Results and Discussion

Phenolic compounds synthesized for chemical defense in natural products are associated with many bioactive properties, such as antioxidant, anti‐inflammatory, antidiabetic, and anticancer properties. This has sparked curiosity about the phenolic content of honey. The main objective of this study was to analyze the phenolic profiles of multifloral honey samples obtained from Türkiye. We used the LC‐MS/MS technique to analyze 25 different phenolic compounds in multifloral honey produced in seven different geographical regions of Türkiye. The phenolic content varied by region, and there were statistical differences between them. The highest measured phenolic component was hydroxycinnamic acid in honey samples from Central Anatolia (100.94 ± 12.79 μg/100 g) and Eastern Anatolia (103.60 ± 17.76 μg/100 g) regions, followed by the Marmara (119.32 ± 17.26 μg/100 g), Aegean (147.88 ± 15.99 μg/100 g), and the Black Sea (204.83 ± 33.28 μg/100 g) regions, while vanillic acid was the highest in honey samples from the Mediterranean (114.38 ± 41.52 μg/100 g) and Southeastern Anatolia (148.53 ± 81.72 μg/100 g) regions. The phenolic compounds identified in Turkish honey are presented in Tables 2 and 3, and Figure S1. LC‐MS/MS data revealed 20 different phenolic compounds in Turkish honey, as shown in Tables 2 and 3 and Figure S1. The Eastern Anatolian honey samples had the greatest diversity of phenolic compounds, with 20 different types. In contrast, Aegean and Black Sea honeys had the least diversity, with 16 phenolic compounds.

**TABLE 2:** Composition of phenolic compounds of honey samples by the province of Türkiye (μg/100 g).

Honey samples	1	2	3	4	5	6	7	8	9	10	11	12	13	14	15	16	17	18	19	20
Eastern Anatolia																				
Ağrı	2.49	—	41.5	4.52	—	—	89.28	6.91	—	144.36	—	10.99	6.51	50.28	—	—	—	3.03	1.8	3.13
Ardahan	—	—	46.22	—	9.29	4.5	6.17	8.94	14.5	27.57	—	8.64	1.47	4.32	—	—	—	2.92	2.37	9.86
Ardahan	—	—	33.95	5.62	—	—	2.89	11.19	15.77	7.02	—	8.15	—	—	—	—	4.65	1.42	0.11	9.47
Ardahan	2.34	—	57.16	6.69	7.39	—	6.28	8.58	5.17	43.09	30.41	8.46	—	2.27	—	—	—	2.89	1.63	11.0
Ardahan	2.31	—	35.66	4.85	9.03	4.76	7.03	8.41	26.41	25.68	—	—	1.14	2.41	—	—	—	2.38	1.06	9.75
Bingöl	2.75	—	50.21	3.89	6.21	4.26	1.67	4.71	—	—	—	8.03	1.54	10.82	—	—	—	3.63	2.81	6.62
Bingöl	—	0.64	135.12	5.22	6.95	4.64	12.01	6.56	4.79	14.02	34.92	6.85	0.21	—	—	—	—	0.86	—	—
Bingöl	2.63	—	47.74	5.14	6.09	4.66	126.27	18.43	26.92	108.96	30.02	7.44	—	—	—	—	—	6.37	4.04	—
Bingöl	—	—	44.67	4.85	8.63	—	68.52	12.19	36.3	58.38	—	13.49	1.67	9.96	—	—	—	18.29	15.35	—
Bitlis	—	—	54.7	4.45	5.78	—	17.78	7.21	16.99	33.48	—	9.22	1.73	11.7	—	—	4.79	3.88	3.28	—
Elazığ	—	—	92.45	8.54	—	—	91.04	11.5	—	188.53	—	13.54	0.36	1.89	—	—	—	5.5	5.11	—
Elazığ	—	—	78.63	8.88	47.84	8.48	29.75	11.01	26.62	90.8	—	14.52	15.25	52.77	18.25	19.85	—	2.88	2.13	5.95
Elazığ	—	—	63.61	3.62	11.54	—	75.35	8.02	15.95	108.28	—	15.9	0.15	—	—	—	—	11.7	10.54	—
Elazığ	20.85	—	40.71	7.32	16.71	—	64.7	8.77	6.7	314.31	—	9.82	2.39	23.62	—	—	—	2.45	1.1	7.41
Erzurum	2.64	—	37.22	5.91	—	4.64	56.46	27.69	6.63	549.81	39.18	8.04	4.96	13.64	9.6	28.93	4.8	2.77	2.3	6.36
Erzurum	—	—	53.32	4.52	12.54	—	12.82	12.01	34.37	47.67	—	18.11	12.51	33.58	42.65	17.72	—	2.14	0.78	9.4
Erzurum	—	—	43.29	5.66	20.59	4.54	27.77	8.36	14.52	49.71	30.31	6.85	—	—	—	35.85	—	6.04	5.43	84.6
Erzurum	—	—	53.72	6.28	11.03	—	58.38	7.13	8.31	144.08	26.27	16.42	5.62	46.7	49.49	40.49	—	4.1	2.76	18.74
Erzurum	5.22	—	71.94	8.71	11.06	—	54.33	23.92	10.3	106.08	—	11.87	2.94	21.98	—	—	—	9.09	8.08	5.91
Kars	2.64	—	37.43	5.91	6.3	—	17.51	10.43	28.48	35.78	—	6.91	1.98	10.69	—	—	4.54	4.42	3.29	9.25
Kars	—	—	39.77	6.5	8.93	5	38.08	12.72	10.96	47.96	—	13.58	1.06	8.81	—	—	4.59	27.42	29.08	5.36
Kars	2.48	0.31	47.23	4.52	49.55	4.57	49.95	8.31	26.46	55.34	—	14.55	2.68	14.31	—	—	—	14.82	13.6	4.7
Kars	—	0.32	46.44	4.35	8.57	4.53	31.36	14.92	25.32	56.29	26.09	11.36	2.61	16.69	—	—	4.69	7.96	7.06	—
Kars	—	—	46.26	4.65	4.28	—	33.28	6.72	18.99	32.94	29.11	12.87	0.162	—	—	—	4.48	3.35	2.85	6.71
Kars	2.49	0.65	59.81	4.85	41.99	5.74	16.94	9.47	11.38	140.01	34.13	15.63	2.55	19.08	—	13.97	4.48	4.11	3.89	5.87
Kars	—	—	62.83	8.14	11.46	4.39	36.07	14.14	20.16	120.27	—	8.47	2.2	10.8	—	19.82	4.69	13.65	12.88	9.13
Kars	2.49	—	71.47	5.86	10.65	5.07	14.22	6.98	3.64	27.43	—	8.97	1.15	9.13	—	28.8	—	4.65	3.82	7.24
Malatya	2.67	0.44	37.82	3.62	13.38	4.41	31.26	6.23	3.54	114.24	—	8.99	0.48	6.37	—	—	—	3.09	2.83	5.06
Malatya	—	0.45	53.06	6.08	12.13	—	106.23	8.35	9.84	184.25	26.74	12.99	—	—	—	15.65	5.43	14.99	12.12	4.57
Tunceli	19.92	—	56.43	7.19	15.85	—	60.28	10.59	9.85	171.87	—	9.2	3.37	21.34	—	42.44	—	3.06	1.86	8.58
Tunceli	25.46	—	54.28	6.91	19.11	—	85.93	32.84	20.64	204.09	—	6.85	1.12	1.78	—	—	4.59	2.42	1.28	—
Van	—	—	51.19	4.95	19.71	4.53	9.71	4.93	3.44	40.03	—	8.16	2.66	—	—	—	—	12.09	10.27	—
Van	—	0.51	51.53	4.94	6.79	—	90.96	6.56	4.88	58.21	—	13.54	1.14	6.61	—	—	—	6.76	5.02	—
Van	2.31	—	39.35	3.23	8.74	4.31	86.65	8.91	5.2	62.48	—	17.53	0.7	5.12	—	—	—	7.09	6.18	—
Van	2.36	—	72.89	5.66	15.56	4.62	11.55	13.42	6.74	109.4	—	9.22	0.55	—	—	—	4.48	6.62	5.97	—
Mediterranean																				
Adana	3.01	—	517.09	12.96	12.91	—	18.88	5.49	3.64	22.9	—	9.16	1.23	—	—	—	—	5.96	4.79	5.36
Adana	3.06	—	41.42	3.57	16.67	—	12.81	7.85	15.2	24.01	—	6.77	—	—	—	—	—	2.97	1.46	12.09
Adana	—	—	49.81	3.18	7.7	5.53	24.12	7.79	11.43	76.13	—	17.26	1.79	14.81	—	17.21	—	4.79	3.37	6.97
Adana	24.99	—	56.75	7.1	25.98	4.54	25.61	9.55	40.41	155.58	—	11.76	—	—	—	—	4.53	5.33	4.5	15.55
Antalya	4.65	—	162.9	—	61.85	8.89	13.36	13.82	10.37	98.26	62.11	—	—	—	—	—	—	34.77	31.7	9.23
Hatay	5.08	—	76.37	9.04	12.84	—	1.753	—	—	—	57.18	13.58	4.81	—	—	—	—	2.31	—	—
Kahramanmaraş	—	—	82.63	9.61	67.46	10.03	22	16.27	7.29	221.84	66.94	17.63	0.99	—	—	—	—	112.43	110	—
Kahramanmaraş	—	—	74.81	15.47	16.08	—	24.09	16.89	16.47	92.84	52.71	12.5	—	—	—	—	—	13.64	11.62	—
Mersin	2.46	—	37.97	4.28	12.41	4.54	6.85	13.88	22.6	31.1	—	18.85	0.43	—	—	—	4.93	25.17	23.79	—
Mersin	4.79	—	79	11.81	10.83	—	9.88	16.14	10.3	53.59	—	14.7	—	—	—	—	9.08	41.74	40.96	—
Mersin	5.23	—	79.42	10.26	75.9	9.23	7.41	15.28	13.33	92.12	—	36.4	—	—	—	—	9.29	196.81	197.7	—
Marmara																				
Balıkesir	4.9	—	75.22	9.88	52.19	9.8	15.21	13.18	16.56	65.77	—	20.17	—	—	—	—	9.07	50.83	45.97	—
Balıkesir	4.84	—	74.09	10.25	14.24	9.81	17.57	19.38	48.52	77.07	53.65	18.02	—	—	—	—	—	53.21	51.38	13.39
Balıkesir	—	—	117.53	7.33	64.12	12.21	24.51	13.3	14.28	107.2	—	21.28	—	—	—	—	—	60.66	58.46	8.68
Balıkesir	5.54	0.54	58.68	16.7	97.66	10.07	30.59	15.24	16.39	119.16	83.64	18.2	1.6	—	—	34.59	9.31	19.73	17.67	11
Balıkesir	—	—	38.91	3.96	7.17	4.49	27.06	8.01	4.85	22.09	34.84	9.04	4.7	39.35	—	20.74	4.54	1.69	0.69	5.21
Balıkesir	5.55	—	72.73	11.63	100.57	11.49	12.91	11.22	10.36	119.71	109.59	13.7	—	—	—	—	—	71.56	71.35	—
Balıkesir	4.75	—	80.66	7.33	109.14	11.18	14.92	12.18	25.23	68.86	54.01	13.7	—	—	—	—	—	77.48	73.76	—
Balıkesir	5.1	—	75.54	10.56	174.75	9.76	40.78	13.03	19.69	198.22	58.03	—	1.69	0.4	—	39.3	—	65.15	58.84	—
Balıkesir	4.78	—	85.9	6.91	34.35	10.02	12.47	19.55	22.28	123.15	80.57	27.84	—	—	—	—	—	26.79	24.35	—
Balıkesir	5.32	—	86.06	12.76	172.42	10.04	15.49	9.57	16.39	96.84	60.96	15.75	—	—	—	—	—	50.49	52.4	—
Balıkesir	4.94	—	82.68	9.71	254.85	11	16.15	10.17	—	162.41	71.21	13.7	—	—	—	—	—	89.18	88.44	—
Balıkesir	10.02	—	36.49	10.77	25.34	13.22	7.01	16.29	10.17	62.71	—	—	1.41	—	—	—	—	1.95	5.01	—
Bursa	—	—	56.09	25.05	24.19	—	6.01	10.03	6.05	19.71	149.41	—	—	—	—	—	9.07	163.01	151.52	—
Çanakkale	4.68	—	640.15	11.82	148.93	10.5	42.28	12.99	10.36	199.72	53.5	15.68	—	—	—	30.03	—	75	66.99	—
Çanakkale	—	—	60.57	9.04	58.99	11.55	29.38	10.42	19.48	132.92	59.06	15.81	—	—	—	—	—	70.22	72.91	8.7
Çanakkale	—	—	84.59	25.38	38.8	10.11	31.11	20.93	10.1	157.48	74.39	14.88	—	—	—	39.62	8.97	20.31	19.73	—
Edirne	5.43	—	108.87	9.91	201.67	10.9	23.06	12.04	7.18	131.52	—	15.85	—	—	—	—	—	39.64	35.31	9.64
Tekirdağ	2.47	0.38	34.36	7.31	19.69	—	139.59	45.22	12.97	345.75	26.24	8.98	8.2	61.41	—	39.55	4.59	9.87	9.27	7.22
Tekirdağ	2.33	0.45	42.91	4.03	11.53	4.6	26.68	7.11	66.52	56.8	—	9.4	—	1.93	—	—	4.54	10.93	10.22	12.2
Black Sea																				
Artvin	—	—	113.95	—	80.84	9.06	42.84	187.64	14.8	268.26	—	25.65	7.94	49.25	—	106.01	—	19.21	15	18.39
Kastamonu	—	—	92.4	15.5	53.19	10.02	20.31	13.28	—	190.56	79.39	24.39	—	—	—	—	—	15.8	15.02	—
Tokat	—	—	43.14	4.09	—	—	45.79	6.8	17.49	155.65	26.47	7.91	9.92	17.25	—	34.19	—	1.69	1.04	—
Aegean																				
İzmir	5.74	—	139.07	11.34	103.67	10.07	41.19	11.39	7.29	174.62	—	16.9	1.99	—	—	—	9.18	71.99	74.36	—
İzmir	5.22	—	78.19	7.96	274.15	9.1	20.74	10.25	25.88	100.26	—	16.96	—	—	—	—	9.2	81.99	81.37	—
İzmir	4.97	—	69.68	12.49	143.43	9.74	14.33	16.12	25.94	154.69	—	13.57	—	—	—	—	—	92.4	95.26	—
İzmir	—	—	76.33	8.7	59.52	9.5	21.32	10.73	7.28	158.16	—	13.67	—	—	—	—	—	43.71	40.93	—
İzmir	5.9	—	86.62	9.57	252.94	9.43	27.96	10.63	19.7	246.92	—	13.58	—	—	—	—	—	83.46	87.01	—
İzmir	4.7	—	84.71	16.8	16.33	—	24.2	12.12	—	115.71	—	—	2.34	5.61	—	—	—	12.44	12.79	—
Kütahya	4.66	—	95.4	8.89	15.78	—	52.28	10.47	—	176.83	—	13.7	2.13	12.59	—	—	9.07	77.48	73.76	—
Muğla	4.98	—	75.3	12.56	90.24	10.68	24.06	12.81	13.26	125.43	—	15.82	—	—	—	—	—	95.67	90.04	—
Muğla	11.08	—	97.25	8.04	177.24	10	13.36	11.4	10.51	61.62	—	12.41	—	—	—	—	9.18	86.57	81.99	—
Muğla	5.06	—	118.07	7.24	55.52	9.86	43.67	10.45	13.42	164.55	—	17.24	0.01	—	—	—	—	69.89	70.88	—
Muğla	—	—	105.45	11.11	—	—	1.35	—	—	—	52.96	—	3.79	—	—	—	—	1.53	—	—
Central Anatolia																				
Ankara	4.66	—	79.13	6.36	106.18	8.9605	15.8	—	10.3	52.63	—	—	—	—	—	—	—	65.19	61.05	—
Afyon	2.38	0.56	24.43	—	8.44	—	101.39	6.61	4.71	130.49	—	9.6	4.3	35.93	—	—	—	5.36	3.57	6.94
Konya	4.96	—	83.27	9.9	—	8.5884	18.32	11.07	11.38	50.12	—	18.11	—	—	—	—	9.06	4.31	1.95	—
Sivas	—	—	73.58	7.24	14.92	8.4917	94.93	14.42	38.15	98.91	—	19.97	0.21	6.25	—	—	9.18	5.23	2.8	—
Sivas	2.77	—	43.18	4	—	4.3644	84.42	7.95	25.33	91.49	—	9.96	2.7	18.32	—	—	4.53	2.29	1.82	—
Sivas	—	—	95.6	9.35	11.9	—	133.96	14.71	32.95	143.5	—	14.68	4.35	20.56	—	—	—	3.35	2.12	—
Sivas	—	—	122.43	9.96	11.55	—	39.92	23	10.07	139.42	61.38	15.73	4.08	19.22	—	—	—	1.41	0.28	—
Southeastern Anatolia																				
Adıyaman	2.37	—	38.25	3.54	8.19	—	20.91	5.48	—	79.7	—	15.35	3.17	16.26	—	—	—	0.93	0.01	5.19
Adıyaman	—	—	38.91	4.35	—	—	7.27	7.36	—	36.83	—	—	—	—	—	—	—	2.95	1.23	6.57
Adıyaman	—	—	74.61	—	—	4.32	17.24	5.11	8.33	49.14	—	9	—	—	—	—	—	8.37	7.43	9.2
Adıyaman	—	—	1123.53	8.89	9.02	—	47.04	10.36	—	60.85	65.8	20	5.41	43.01	—	98.5	9.27	3.15	0.15	—
Adıyaman	2.38	—	37.16	—	—	—	3.72	7.39	—	1.78	—	8.77	—	0.06	—	—	—	0.44	—	4.55
Adıyaman	—	—	65.67	7.24	—	—	4.02	9.69	9.25	12.1	—	13.49	—	—	—	—	—	2.08	0.9	10.45
Adıyaman	—	—	45.77	4.35	4.81	—	1.04	13.83	14.31	8.22	—	8.04	—	—	—	—	—	1.13	0.02	8.08
Batman	—	—	34.55	4.44	—	—	8.78	6.25	11.27	61.9239	26.74	—	0.07	—	—	—	—	2.73	1.72	8.86
Siirt	4.68	—	131.97	7.93	10.75	—	18.59	11.72	13.42	74.63	—	19.04	—	—	—	—	—	6.82	4.82	—
Siirt	—	—	105.31	7.24	23.112	—	25.121	15.91	9.56	134.8	—	15.38	—	—	—	—	—	10.51	7.28	—
Siirt	—	0.7	91.65	4.45	4.24	4.29	17.99	7.33	7.42	40.43	—	8.07	—	—	—	—	4.75	8.12	7.62	—
Siirt	—	—	100.1	8.7	—	9.05	11.94	17.76	13.48	142.81	—	12.41	—	—	—	—	—	4.98	3.41	—
Şanlıurfa	2.62	—	43.43	6.03	18.54	—	6.93	10.9	19.18	30.1	26.46	7.44	0.35	—	—	—	—	4.77	4.28	6.97

*Note:* ^1^Catechin hydrate, ^2^Acetohydroxamic acid, ^3^Vanillic acid, ^4^Resveratrol, ^5^Fumaric acid, ^6^Gallic acid, ^7^Caffeic acid, ^8^Phloridzindihydrate, ^9^Oleuropein, ^10^Hydoxy cinnamic acid, ^11^Ellagic acid, ^12^Silymarin, ^13^Butein, ^14^Naringenin, ^15^Luteolin, ^16^Kaempferol, ^17^Curcumin, ^18^Hydroxybenzoic acid, ^19^Salicylic acid, ^20^Quercetin, —: <limit of detection.

**TABLE 3:** List the phenolic components of the honey samples by geographical regions of Türkiye (μg/100 g) (mean ± SE).

Regions								
Phenolic compounds	Eastern Anatolia (*n* = 35)	Mediterranean (*n* = 11)	Marmara (*n* = 19)	Black Sea (*n* = 3)	Aegean (*n* = 11)	Central Anatolia (*n* = 7)	Southeastern Anatolia (*n* = 13)	TÜRKİYE (*n* = 99)
Catechin hydrate	6.122 ± 1.871 <LoD = 18	6.662 ± 2.472 <LoD = 3	5.050 ± 0.467 <LoD = 5	<LoD	5.81 ± 7.673 <LoD = 2	3.696 ± 0.507 <LoD = 3	3.017 ± 0.558 <LoD = 9	5.487 ± 4.721 <LoD = 43
Acetohydroxamic acid	0.474 ± 0.117^b^ <LoD = 28	<LoD	0.462 ± 0.70^b^ <LoD = 16	<LoD	<LoD	0.560 ± 0.00^b^ <LoD = 6	0.706 ± 0.00^a^ <LoD = 12	0.498 ± 0.168 <LoD = 87
Vanillic acid	54.56 ± 3.26	114.382 ± 41.525	100.639 ± 30.425	83.166 ± 20.954	93.284 ± 6.319	74.523 ± 10.631	148.536 ± 81.722	88.972 ± 130.66
Resveratrol	5.651 ± 0.225^c^ <LoD = 1	8.7301 ± 5.00^ab^ <LoD = 1	11.075 ± 1.330^a^	9.8005 ± 0.701^ab^ <LoD = 1	10.431 ± 0.004^a^	7.806 ± 0.820^abc^ <LoD = 1	6.215 ± 0.555^bc^ <LoD = 2	7.938 ± 4.382 <LoD = 6
Fumaric acid	14.26 ± 0.008^c^ <LoD = 4	29.153 ± 7.783^b^	84.77 ± 16.800^ab^	67.02 ± 13.800^b^ <LoD = 1	118.90 ± 29.00^a^ <LoD = 1	30.648 ± 15.500^b^ <LoD = 2	11.240 ± 2.627^c^ <LoD = 6	46.211 ± 61.659 <LoD = 14
Gallic acid	4.874 ± 0.227^c^ <LoD = 17	7.131 ± 1.686^b^ <LoD = 5	10.049 ± 0.552^a^ <LoD = 2	9.545 ± 0.484^a^ <LoD = 1	9.804 ± 0.1170^a^ <LoD = 3	7.601 ± 0.839^b^ <LoD = 3	5.891 ± 0.58^c^ <LoD = 10	7.706 ± 4.379 <LoD = 41
Caffeic acid	43.676 ± 5.754^ab^	15.166 ± 2.478^b^	28.045 ± 6.624^b^	36.317 ± 8.048^b^	25.864 ± 4.469^b^	69.825 ± 14.891^a^	14.665 ± 3.401^b^	33.345 ± 31.018
Phloridzindihydrate	11.063 ± 1.042^b^	12.301 ± 1.723^b^ <LoD = 1	14.734 ± 1.906^b^	69.245 ± 59.0308^a^	11.641 ± 1.173^b^ <LoD = 1	12.964 ± 2.041^b^ <LoD = 1	9.934 ± 4.009^b^	13.763 ± 18.752 <LoD = 3
Oleuropein	15.000 ± 1.710^b^ <LoD = 3	15.104 ± 3.406^ab^ <LoD = 1	18.750 ± 3.650^a^ <LoD = 1	16.150 ± 1.35^ab^ <LoD = 1	15.410 ± 2.68^ab^ <LoD = 3	18.990 ± 4.270^a^	11.810 ± 1.23^c^ <LoD = 4	15.851 ± 11.375 <LoD = 13
Hydoxy cinnamic acid	103.605 ± 17.761^bc^ <LoD = 1	86.842 ± 20.582^bc^ <LoD = 1	119.325 ± 17.266^bc^	204.831 ± 33.281^a^	147.883 ± 15.999^ab^ <LoD = 1	100.943 ± 12.799^bc^	56.412 ± 12.192^c^	106.161 ± 82.059 <LoD = 2
Ellagic acid	30.721 ± 0.340^d^ <LoD = 25	59.736 ± 13.562^ab^ <LoD = 7	69.227 ± 3.656^a^ <LoD = 5	59.23 ± 26.400^ab^ <LoD = 1	52.965 ± 0.00^b^ <LoD = 10	61.384 ± 0.00^a^ <LoD = 6	39.700 ± 13.100^c^ <LoD = 10	52.988 ± 29.989 <LoD = 64
Myricetin	<LoD	<LoD	<LoD	<LoD	<LoD	<LoD	<LoD	<LoD
Protocatechuic acid	<LoD	<LoD	<LoD	<LoD	<LoD	<LoD	<LoD	<LoD
Silymarin	11.038 ± 0.581^c^ <LoD = 1	15.866 ± 2.875^ab^ <LoD = 1	15.755 ± 1.210^ab^ <LoD = 3	19.322 ± 5.717^a^	14.878 ± 0.615^bc^ <LoD = 2	14.681 ± 1.455^bc^ <LoD = 1	12.458 ± 1.373^bc^ <LoD = 2	13.517 ± 6.368 <LoD = 10
2‐Hyroxy1,4nph	<LoD	<LoD	<LoD	<LoD	<LoD	<LoD	<LoD	<LoD
Butein	2.766 ± 0.624^b^ <LoD = 5	1.854 ± 0.652^b^ <LoD = 6	3.523 ± 1.318^b^ <LoD = 14	8.93 ± 5.985^a^ <LoD = 1	2.053 ± 0.604^b^ <LoD = 6	3.128 ± 0.645^b^ <LoD = 2	2.253 ± 1.263^b^ <LoD = 9	2.905 ± 0.706 <LoD = 43
Naringenin	16.030 ± 2.872^ab^ <LoD = 9	14.81 ± 0.00^b^ <LoD = 10	25.776 ± 14.906^ab^ <LoD = 15	33.258 ± 15.999^a^ <LoD = 1	9.099 ± 3.491^b^ <LoD = 9	20.062 ± 3.858^ab^ <LoD = 2	19.778 ± 12.523^ab^ <LoD = 10	18.118 ± 13.904 <LoD = 56
Luteolin	30.002 ± 16.540 <LoD = 31	<LoD	<LoD	<LoD	<LoD	<LoD	<LoD	30.002 ± 16.540 <LoD = 95
Kaempferol	26.360 ± 3.310^c^ <LoD = 25	17.209 ± 3.669^c^ <LoD = 10	33.98 ± 0.070^b^ <LoD = 13	70.100 ± 35.000^a^ <LoD = 1	<LoD	<LoD	98.506 ± 0.00^a^ <LoD = 12	36.168 ± 18.145 <LoD = 61
Curcumin	4.689 ± 0.075^c^ <LoD = 23	6.9621 ± 0.892^b^ <LoD = 7	7.161 ± 0.921^b^ <LoD = 12	<LoD	9.162 ± 0.028^a^ <LoD = 7	7.600 ± 1.006^b^ <LoD = 4	7.010 ± 2.260^b^ <LoD = 11	6.491 ± 3.302 <LoD = 64
Thymoquinone	<LoD	<LoD	<LoD	<LoD	<LoD	<LoD	<LoD	<LoD
Alizarin	<LoD	<LoD	<LoD	<LoD	<LoD	<LoD	<LoD	<LoD
Hydroxybenzoic acid	6.543 ± 0.960^b^	40.543 ± 8.381^a^	50.410 ± 8.907^a^	12.238 ± 5.363^b^	65.198 ± 9.656^a^	12.452 ± 7.627^b^	4.387 ± 0.889^b^	25.564 ± 36.549
Salicylic acid	5.671 ± 0.991^c^ <LoD = 1	43.001 ± 19.455^b^ <LoD = 1	48.124 ± 8.421^b^	10.358 ± 4.656^c^	70.845 ± 7.990^a^ <LoD = 1	10.515 ± 7.301^c^	3.240 ± 0.866^c^ <LoD = 1	25.150 ± 36.116 <LoD = 4
Quercetin	11.076 ± 3.406^ab^ <LoD = 12	9.845 ± 2.571^b^ <LoD = 6	9.509 ± 0.941^b^ <LoD = 11	18.399 ± 0.00^a^ <LoD = 2	<LoD	6.940 ± 0.004^c^ <LoD = 6	7.488 ± 0.718^b^ <LoD = 5	10.116 ± 9.419 <LoD = 42

*Note:* Values with different superscripts letters (a–e) in the same column are statistically different *p* < 0.05.

Abbreviations: <LoD, limit of detection; *n*, number of samples.

Although it is difficult to identify the specific polyphenols that reliably indicate particular honey varieties, it is remarkable that the presence of hydroxybenzoic acid, vanillic acid, and caffeic acid was detected in all multifloral honey samples obtained from different geographical regions of Türkiye in this study. These results align with those of Kıvrak and Kıvrak (2017), who examined the phenolic profile of Turkish honey. Caffeic and vanillic acids, which are derivatives of hydroxycinnamic acid from the phenolic acid subgroup, have been detected in various honey samples worldwide (Lawag et al. 2022). Caffeic acid, also known as 3,4‐dihydroxycinnamic acid, is one of the most prevalent hydroxycinnamic acid derivatives found in honey (Moore et al. 2025).

Examining the frequency of phenolic compounds in honey samples revealed that vanillic acid, caffeic acid and hydroxybenzoic acid were present in all samples. The next most prevalent compounds were hydroxycinnamic acid (97%), phloridzin dihydrate (97%), salicylic acid (95%), resveratrol (94%), silymarin (89%) and oleuropein (86%). Fumaric acid (85%), gallic acid (58%), butein (56%), catechin hydrate (56%), quercetin (46%), naringenin (43%), kaempferol (39%), curcumin (36%), ellagic acid (35%), acetohydroxamic acid (12%), and luteolin (4%). Alizarin, myricetin, protocatechuic acid, thymoquinone and 2‐hydroxy‐1,4‐naphthoquinone were not detected. Similarly, Kıvrak and Kıvrak (2017) reported that the myricetin levels in various honey samples from different regions and provinces in Türkiye were below the LoD.

**FIGURE 2:**
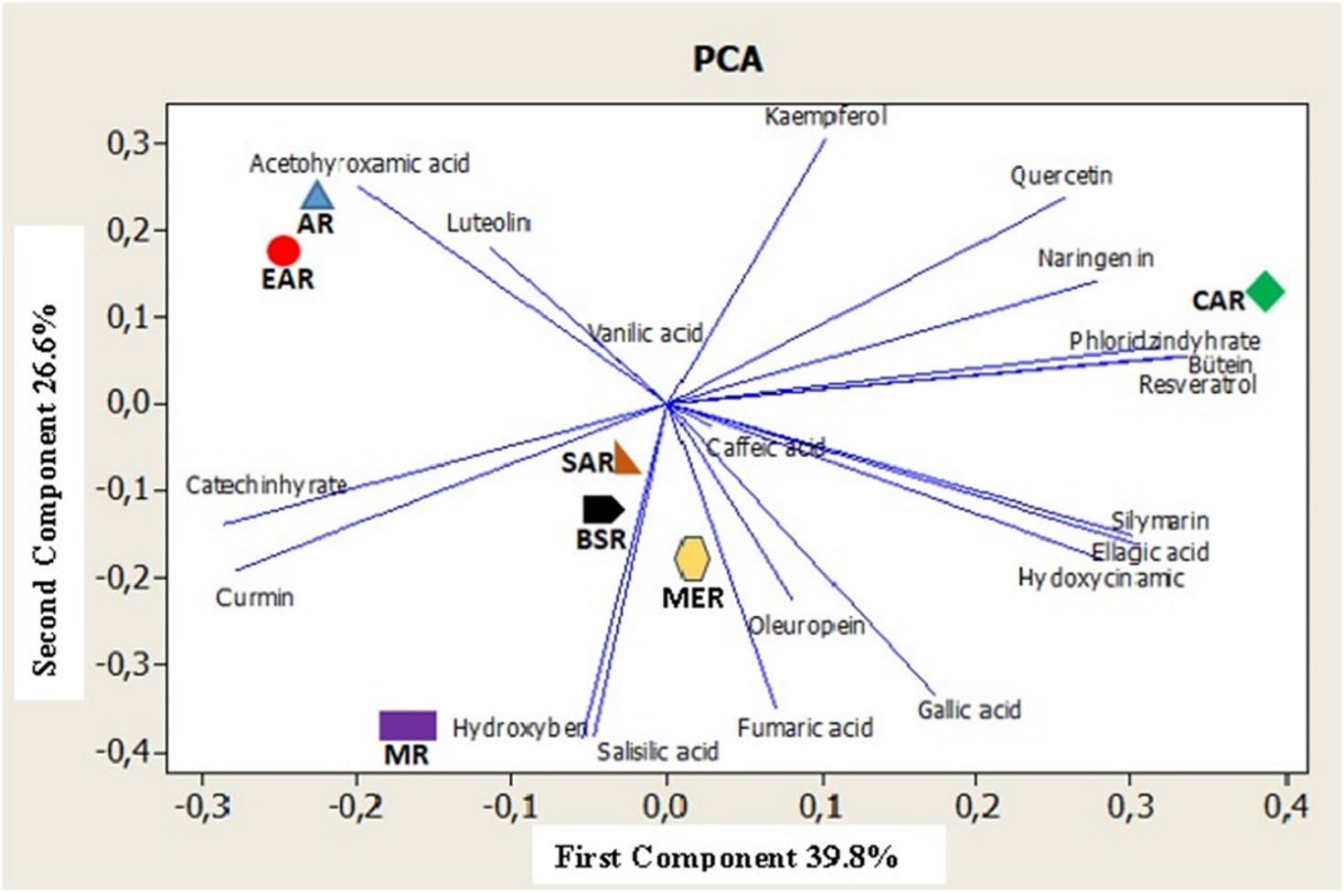
The results of the principal component analysis PCA with honey. AR, Aegean Region; BSR, Black Sea Region; CAR, Central Anatolia Region; EAR, Eastern Anatolia Region; MER, Mediterranean region; MR, Marmara Region; SAR, Southeastern Anatolia Region.

The results of the principal component analysis (PCA) performed on the honey samples are presented in Figure 2. The PCA analysis indicates that the combined variance accounts for 39.8% of the first component (PC1) and 26.6% of the second component (PC2). The first two principal components (PC1 and PC2) together describe 66.4% of the total variance in the data set, with PC1 exhibiting the highest variance. When PC1 and PC2 were analyzed for the honey samples, it was observed that the Central Anatolia Region exhibited positive attributes, while the other regions exhibited negative attributes in PC1. Meanwhile, the Eastern Anatolia Region, the Central Anatolia Region, and the Aegean Region are characterized as positive in PC2, while other regions are negative. Examining previous studies on the phenolic content of Turkish honeys (Can et al. 2015; Kıvrak and Kıvrak 2017; Sıcak et al. 2021; Taş‐Küçükaydın et al. 2023; Çayan et al. 2023; Kolaylı et al. 2024) revealed that most of the samples were unifloral honeys, hence their inclusion in the discussion.

### 3.1 Eastern Anatolia Honey

In addition to the three phenolic acids detected in all samples in the study (vanillic, caffeic and hydroxybenzoic), phloridzin dihydrate was also present in all honeys from the Eastern Anatolian region (see Table 3). The three most abundant phenolic compounds were hydroxycinnamic acid, vanillic acid and caffeic acid, in that order. While there was no statistically significant difference between regions, Eastern Anatolian honey had the lowest vanillic acid value. The Eastern Anatolian region had the highest frequency of butein (85.7%) and naringenin (74.2%) in honey samples. Butein, a flavonol with strong antioxidant properties, is found in various plants, including some members of the Asteraceae family (Hamdani et al. 2025). Naringenin, together with luteolin and gallic acid, is being evaluated as a potential marker for lavender honeys (Jaśkiewicz et al. 2025). Luteolin was only detected in honey collected from four provinces in this region, three of which were in Erzurum province. The presence of luteolin in honey has been associated with coolibah (*Eucalyptus coolabah*) honey (Moore et al. 2025). Šarić et al. (2020) reported that Croatian acacia honeys also contain luteolin. A study by Küçükaydın et al. (2023) investigating the phenolic content of *Astragalus* honeys from provinces within the Eastern Anatolia region (Diyarbakır, Elazığ, Erzincan, Erzurum and Tunceli) reported the presence of gallic acid, vanillic acid and hydroxybenzoic acid in all samples. The study found an average of 51% of Eastern Anatolian honeys contained gallic acid. Myricetin was not present in any of the honey samples analyzed in either study. Phenolic compounds are the most effective molecules for confirming the botanical source of honey, as they are transferred to honey via nectar (Ferreres et al. 1996). Kaempferol has been reported as a potential marker for rosemary honey (Tomás‐Barberán et al. 2001). However, this flavonol can also be obtained from other floral sources, including sunflower and fennel nectar (Shen et al. 2019). In this study, kaempferol was detected in 55.5% of the honey samples, most of which were from Eastern Anatolia.

### 3.2 Mediterrenian Honey

The dominant phenolics in Mediterranean honey were vanillic acid (114.38 ± 41.52 μg/100 g), hydroxy cinnamic acid (86.84 ± 20.58 μg/100 g), and ellagic acid (59.73 ± 13.56 μg/100 g). Studies conducted on Mediterranean honey (Kıvrak and Kıvrak 2017; Söğüt and Seydim 2020; Çayan et al. 2023; Kolaylı et al. 2024) have shown that the frequency and quantity of phenolic substances in honey can differ considerably from those reported in this study. Similar to the results of our study, it has been reported that protocatechuic acid, myricetin, and luteolin could not be detected in lavender (*Lavandula* Spp.) honey obtained from the Isparta province in the Mediterranean region. However, contrary to the findings of this study, Kolaylı et al. (2024) reported that caffeic acid, ellagic acid, and resveratrol were below the detection limits in all honey samples. Çayan et al. (2023) reported the presence of gallic acid (75.71–84.47 μg/g), protocatechuic acid (0.01 μg/g), catechin hydrate (0.01 μg/g), hydroxybenzoic acid (1.38 μg/g), and luteolin (3.71–3.74 μg/g) and kaempferol (0.01 μg/g) were detected in all five cotton honey samples obtained from the Adana province. Vanillic acid and quercetin, however, were below the limit of detection. Söğüt and Seydim (2020) found gallic acid values of 3.23–4.21 μg/g and vanillic acid values of 3.72–4.15 μg/g in four and three of the seven honey samples from seven provinces in this region, respectively. Kıvrak and Kıvrak (2017) investigated the presence of various phenolic compounds in different types of honey sourced from various regions of Turkey. They reported finding quercetin, resveratrol, and catechin hydrate at concentrations below the detection limit in citrus, acacia, lavender, and cotton honeys from provinces in the Mediterranean region. These results differ from those of our study. Another study conducted on the opposite coast of the Mediterranean with multifloral honeys from Northern Cyprus reported that protocatechuic acid, luteolin, and myricetin were below the detection limit in all but one sample (similar to our study's results in the Mediterranean region; Uçar et al. 2023). The frequency and quantity of the other examined phenolic compounds (caffeic acid, ellagic acid, resveratrol, quercetin, and curcumin) varied considerably. The phenolic compound with the most obvious difference was caffeic acid. This is because it was detected in all samples of this study (15.16 ± 2.47 μg/100 g) but not in Northern Cyprus honeys.

### 3.3 Marmara Honey

The most notable characteristic of honey from the Marmara Region is its high concentration of gallic acid (89.4%) and ellagic acid (73.6%), which is higher than in other regions. These acids are among the most prevalent phenolic acids and are reported to originate from plant sources of honey that are rich in bioactive compounds, such as *Eucalyptus grandis* (Jaśkiewicz et al. 2025; Hameed et al. 2024; Santos et al. 2012). Some phenolic compounds found in honey have been identified as chemical biomarkers. Gallic acid was identified in lavender honey, while ellagic acid was found in heather honey (Jaśkiewicz et al. 2025). Gallic acid is also classified as a phenolic compound found in citrus fruits (Tounsi et al. 2011). Resveratrol, a natural phenolic compound found in foods such as grapes, peanuts, and blueberries, has been shown to be effective in preventing and managing cancer, cardiovascular disease, neurodegenerative disease, and obesity. It also has anti‐aging, anti‐inflammatory, antioxidant, and immunomodulatory properties (Zhou et al. 2021; Meng et al. 2020). The highest resveratrol values (11.07 ± 1.33 μg/100 g) were found in honeys from this region. In research conducted by Küçükaydın et al. (2023) in the Marmara region, involving chestnut honey, similar honey vanillic acid values were observed, with notably higher levels of gallic acid and quercetin compared to this study.

### 3.4 Black Sea Honey

Phloridzin dihydrate is considered a promising antidiabetic compound due to its ability to decrease glucose absorption in the gastrointestinal system (Londzin et al. 2018). Silymarin is a component of the milk thistle seed ( *Silybum marianum* ), which is known for its hepatoprotective properties (Yıldız et al. 2013). Honeys from the Black Sea region, which were included in the study alongside three other honey samples, were found to have remarkable levels of phloridzin dihydrate (69.24 ± 59.03 μg/100 g), hydroxycinnamic acid (204.83 ± 33.28 μg/100 g), silymarin (19.32 ± 5.71 μg/100 g), butein (8.93 ± 5.98 μg/100 g), and naringenin (33.25 ± 15.99 μg/100 g). This is because the values for these phenolic compounds are highest in the honeys of this region compared to all others. However, catechin hydrate was not detected in these honeys, in contrast to those from other regions. Saral (2023) analyzed 19 phenolic compounds in chestnut honey collected from Artvin Province in Türkiye's Black Sea Region. Consistent with our findings, luteolin could not be detected. However, in contrast to the results of this study, catechin hydrate was the predominant flavonoid in the chestnut honey samples. Kıvrak and Kıvrak (2017) reported that the most abundant phenolic compounds in two honey samples from the same region were caffeic acid, hydroxybenzoic acid, and vanillic acid. Except for caffeic acid, these results are similar to ours. Another study (Küçükaydın et al. 2023) that examined chestnut honey from the Black Sea region produced results that differed from ours in that it reported high vanillic and gallic acid values. Proto catechic acid (0.01–0.38 μg/g) was detected in all honeys, while hydroxycinnamic acid and quercetin were below the detection limit.

### 3.5 Aegean Honey

This study found that the Black Sea and Aegean regions had the least diverse honey phenolic content, containing 16 different phenolic compounds. The most abundant phenolic compounds were similar to those found in the Marmara region, namely hydroxybenzoic acid, vanillic acid, and fumaric acid. The hydroxybenzoic acid values of honey from the Aegean, Mediterranean, and Marmara regions, which have a Mediterranean climate, are statistically similar and higher than those of other regions. This suggests that the climate may positively affect the hydroxybenzoic acid content of honey. Honey with the highest levels of fumaric acid, curcumin, salicylic acid, and hydroxybenzoic acid came from this region. Studies on the phenolic content of various honeys from this region have reported the inability to detect resveratrol and quercetin in honeys, contrary to our findings (Sıcak et al. 2021; Kıvrak and Kıvrak 2017). High concentrations of fumaric and salicylic acids have also been reported in pine and thyme honeys (Sıcak et al. 2021). A study of blossom and pine honeys from the Muğla province reported that naringenin, myricetin, quercetin, kaempferol, resveratrol, and vanillic acid levels in honey samples were below the detection limit. While the honey caffeic acid values in this study were similar to those in our study, the gallic acid values were higher (Çobanoğlu et al. 2023).

### 3.6 Central Anatolia Honey

Oleuropein is one of the main phenolic compounds found in olive oil. It is widely used as an anticancer agent among various natural compounds discovered in recent decades (Omar 2010). Unlike honey from other regions, oleuropein was detected in all honey samples from Central Anatolia, where the highest levels of oleuropein (18.99 ± 4.27 μg/100 g) and caffeic acid (69.82 ± 14.89 μg/100 g) were recorded. The three most abundant phenolic compounds in the Central Anatolian honeys were hydroxycinnamic acid, vanillic acid, and caffeic acid, which are similar to those in Eastern Anatolian honeys. Can et al. (2015) reported that gallic acid, protocatechuic acid, vanillic acid, quercetin and kaempferol could not be detected in multifloral honey samples obtained from various provinces in different geographical regions of Türkiye, including Central Anatolia, and the average level of caffeic acid was 2.03 ± 16.72 μg/g. Kıvrak and Kıvrak (2017) reported that quercetin, resveratrol, catechin hydrate, and myricetin were below the detectable limit in cedar, sunflower, and pumpkin flower honeys obtained from the Central Anatolia region. They also detected luteolin and kaempferol in these honeys, which were not present in our study. Furthermore, the mean levels of hydroxybenzoic acid, vanillic acid, gallic acid, caffeic acid, and naringenin were higher than those in our study.

### 3.7 Southeastern Anatolia Honey

A total of 13 honey samples were included in the study, all of which originated from the provinces of Adıyaman, Batman, Siirt, and Şanlıurfa in the Southeastern Anatolia region. Compared to those from other regions, these honeys had the highest vanillic acid values. However, they had the lowest values for fumaric acid, caffeic acid, oleuropein, hydroxycinnamic acid, hydroxybenzoic acid, and salicylic acid. Additionally, the frequency of gallic acid in honey samples from this region was the lowest (23%). Kaempferol was only detected in one sample from Adıyaman province. In a study by Kolaylı et al. (2023) on *Nigella sativa* (black cumin) honeys, samples from Kilis province were reported to have significantly higher levels of gallic acid, ellagic acid, and quercetin than Southeastern Anatolia multifloral honey samples in our study. *N. sativa* honeys collected from provinces in Mediterranean (Burdur) and Central Anatolian (Kayseri) regions also showed high levels of these phenolic compounds, and contrary to our study, myricetin was detected. Çayan et al. (2023) stated in their study on cotton honey obtained from the provinces of Adana and Şanlıurfa, that catechin hydrate, vanillic acid, hydroxybenzoic acid, quercetin, luteolin, and kaempferol were present in varying amounts in different honey varieties. The results of this study differ considerably from those of the researchers, except that luteolin and protocatechuic acid were not detected in honey samples from Southeastern Anatolian provinces in either study.

When the total phenolic content of Turkish honeys was evaluated, the phenolic compounds found in the highest amounts were hydroxy cinnamic acid (106.16 ± 82.05 μg/100 g), vanillic acid (88.97 ± 130.66 μg/100 g), ellagic acid (52.98 ± 29.98 μg/100 g), and fumaric acid (46.21 ± 61.65 μg/100 g), respectively. Studies on multifloral honeys worldwide have reported that gallic acid, caffeic acid, and vanillic acid were the predominant phenolic acids in Indian multifloral honey (Devi et al. 2018). Meanwhile, ellagic and vanillic acids are the main phenolic acids in Serbian honey from the Vojvodina and Zlatibor regions (Gašić et al. 2014). In Italian multifloral honey, gallic and caffeic acids exhibited the highest values among phenolic acids (Perna et al. 2013). In Romanian honeys, protocatechuic acid and myricetin were detected, in contrast to the results of our study, while high concentrations of gallic acid, vanillic acid, caffeic acid, quercetin, and kaempferol were reported (Pauliuc et al. 2020).

## 4 Conclusion

This study reports the results of screening 25 phenolic compounds in multifloral honey originating from seven different geographical regions of Türkiye using the LC‐MS/MS technique. The study revealed that regional diversity significantly affects the chemical composition of honey and determined that the most abundant natural phenolic compounds in honey were hydroxy cinnamic, vanillic, ellagic, and fumaric acids. However, the rankings varied by region. Due to its geographical location, Türkiye is a country that attracts attention with both its honey diversity and production amount, and honey is harvested three seasons a year. The findings of this study demonstrate that geographical regions also influence the diversity of honey's phenolic content and could be employed as a parameter in the classification of honey within industries such as beekeeping, nutrition, cosmetics, and pharmaceuticals.

## Author Contributions

**Nilgün Paksoy:** conceptualization, funding acquisition, investigation, methodology, formal analysis, data curation, writing – original draft, writing – review and editing. **Hisamettin Durmaz:** conceptualization, funding acquisition, investigation, methodology, formal analysis, data curation, writing – original draft, writing – review and editing. **Serap Kılıç Altun:** conceptualization, funding acquisition, investigation, methodology, formal analysis, data curation, writing – original draft, writing – review and editing. **Mehmet Emin Aydemir:** investigation, methodology, formal analysis, visualization, writing – review and editing.

## Funding

This research was funded by Harran University Harran Scientific Research Project Department with project number 18027.

## Conflicts of Interest

The authors declare no conflicts of interest.

## Supporting information


**Figure S1:** fsn371555‐sup‐0001‐FigureS1.jpeg.

**Table S1:** Analytical method validation parameters that belong to the LC‐MS/MS method.

## Data Availability

The authors have nothing to report.
