# Supplementary figures and images for: Profiling of the Endogenous Phenolic Contents of Multifloral Honey From Different Geographical Origins in Türkiye by LC‐MS/MS
Source: Food Sci Nutr. 2026 Feb 19;14(2):e71555. doi: 10.1002/fsn3.71555 (PMC12920703; doi:10.1002/fsn3.71555)

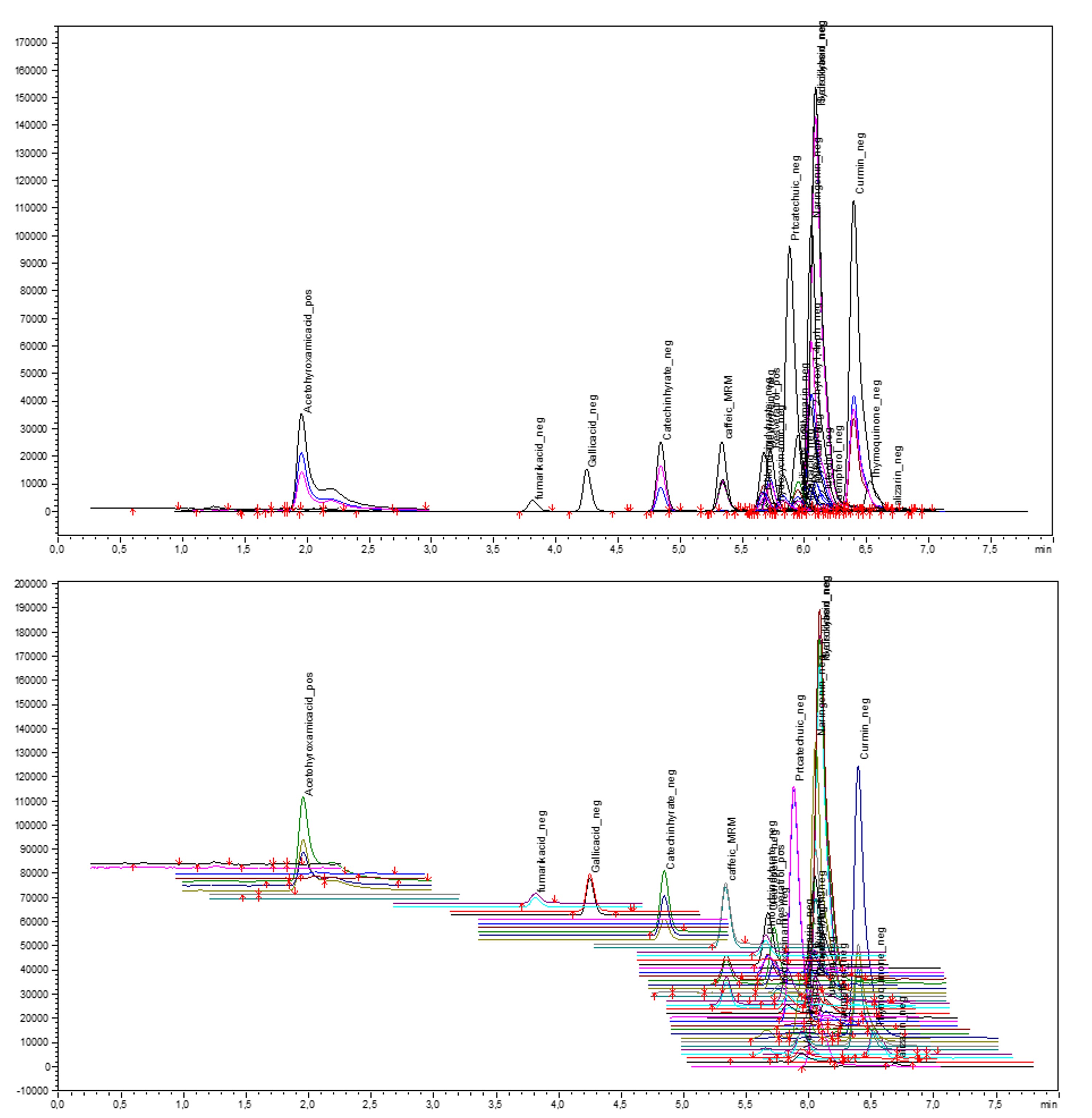

Supplement: Supplementary file 1 — Figure S1: fsn371555‐sup‐0001‐FigureS1.jpeg. [file FSN3-14-e71555-s002.jpeg]
