# Supplementary material for: Profiling of the Endogenous Phenolic Contents of Multifloral Honey From Different Geographical Origins in Türkiye by LC‐MS/MS
Source: Food Sci Nutr. 2026 Feb 19;14(2):e71555. doi: 10.1002/fsn3.71555 (PMC12920703; doi:10.1002/fsn3.71555)
Supplement: Supplementary file 2 — Table S1: Analytical method validation parameters that belong to the LC‐MS/MS method. [file FSN3-14-e71555-s001.docx]

| **Supplementary Table 1.** Analytical method validation parameters that belong to the LC-MS/MS method | | | | | |
| --- | --- | --- | --- | --- | --- |
| Standards | ESI ION MODE | MRM | Retention time | R2 | Equation |
| Quercetin | negative | 301.1>151 | 6.091 | 0.9990363 | Y=(13.7831)X+(-146.951) |
| Acetohydroxamic acid | positive | 76.10>43.10 | 1.986 | 0.999363 | Y = (150.982)X + (23.1833) |
| Catechin hydrate | negative | 291.10>139.00 | 4.958 | 0.9991201 | Y = (79.2933)X + (-2406.22) |
| Vanillic acid | positive | 168.80>93.00 | 6.026 | 0.9976425 | Y = (48.0522)X + (-876.904) |
| Resveratrol | positive | 229.10>135.00 | 5.713 | 0.9978816 | Y = (46.4361)X + (-1314.61) |
| Fumaric acid | negative | 115.20>71.00 | 3.674 | 0.9989117 | Y = (20.2986)X + (-762.592) |
| Gallic acid | negative | 169.20>125.00 | 4.134 | 0.998971 | Y = (65.3835)X + (-2699.84) |
| Caffeic acid | negative | 179.20>135.00 | 5.283 | 0.9956162 | Y = (124.785)X + (-487.132) |
| Phloridzin dihydrate | negative | 435.00>273.10 | 5.646 | 0.9986845 | Y = (33.4069)X + (-1396.90) |
| Oleuropein | negative | 539.10>377.20 | 5.643 | 0.9989262 | Y = (25.9240)X + (-558.916) |
| 4-Hydroxycinnamic acid | negative | 163.20>119.00 | 5.738 | 0.9949564 | Y = (13.1516)X + (717.421) |
| Ellagic acid | negative | 300.90>145.10 | 5.895 | 0.9995757 | Y = (5.25903)X + (-1167.31) |
| Myricetin | negative | 317.10>150.90 | 5.858 | 0.9992188 | Y = (37.0934)X + (2684.23) |
| Protocatechuic acid | negative | 181.20>108.00 | 5.875 | 0.9943057 | Y = (526.954)X + (23026.1) |
| Silymarin | negative | 481.00>301.00 | 5.978 | 0.9947323 | Y = (31.9969)X + (-1823.79) |
| 2-Hydroxy-1.4-naphthoquinone | negative | 173.20>144.90 | 6.058 | 0.9971801 | Y = (203.469)X + (29033.1) |
| Butein | negative | 271.10>135.00 | 6.084 | 0.9992028 | Y = (49.3543)X + (367.917) |
| Naringenin | negative | 271.10>150.90 | 6.104 | 0.9955044 | Y = (317.241)X + (33733.3) |
| Luteolin | negative | 285.20>132.90 | 6.19 | 0.9976786 | Y = (34.6668)X + (3721.79) |
| Kaempferol | negative | 285.10>116.90 | 6.288 | 0.9993608 | Y = (2.63905)X + (-206.494) |
| Curcumin | negative | 367.00>149.00 | 6.516 | 0.9966966 | Y = (227.706)X + (-10111.1) |
| Thymoquinone | negative | 164.20>149.00 | 6.632 | 0.9991933 | Y = (60.4553)X + (2285.92) |
| Alizarin | negative | 239.20>210.90 | 6.8 | 0.998357 | Y = (3.97487)X + (1614.23) |
| Hydroxybenzoic acid | negative | 137.20>93.00 | 6.13 | 0.9987579 | Y = (735.804)X + (-498.102) |
| Salicylic acid | negative | 137.20>93.00 | 6.104 | 0.9989762 | Y = (746.369)X + (6072.41) |

**ESI:** Electrospray Ionization**MRM:** Multiple Reaction Monitoring **R^2^:** Demonstrates the explanatory power of a linear model
